# Molecular oncology focus - Is carcinogenesis a 'mitochondriopathy'?

**DOI:** 10.1186/1423-0127-17-31

**Published:** 2010-04-25

**Authors:** Anna M Czarnecka, Jerzy S Czarnecki, Wojciech Kukwa, Francesco Cappello, Anna Ścińska, Andrzej Kukwa

**Affiliations:** 1 Laboratory of Molecular Oncology, Department of Oncology, Military Institute of the Health Services, Warsaw, Poland; 2 Department of Knowledge Management, Faculty of Management, University of Lodz, Lodz, Poland; 3 Department of Otolaryngology, Czerniakowski Hospital, Medical University of Warsaw, Warsaw, Poland; 4 Human Anatomy Section, Department of Experimental Medicine, University of Palermo, Palermo, Italy

## Abstract

Mitochondria are sub-cellular organelles that produce adenosine triphosphate (ATP) through oxidative phosphorylation (OXPHOS). As suggested over 70 years ago by Otto Warburg and recently confirmed with molecular techniques, alterations in respiratory activity and in mitochondrial DNA (mtDNA) appear to be common features of malignant cells. Somatic mtDNA mutations have been reported in many types of cancer cells, and some reports document the prevalence of inherited mitochondrial DNA polymorphisms in cancer patients. Nevertheless, a careful reanalysis of methodological criteria and methodology applied in those reports has shown that numerous papers can't be used as relevant sources of data for systematic review, meta-analysis, or finally for establishment of clinically applicable markers.

In this review technical and conceptual errors commonly occurring in the literature are summarized. In the first place we discuss, why many of the published papers cannot be used as a valid and clinically useful sources of evidence in the biomedical and healthcare contexts. The reasons for introduction of noise in data and in consequence - bias for the interpretation of the role of mitochondrial DNA in the complex process of tumorigenesis are listed. In the second part of the text practical aspects of mtDNA research and requirements necessary to fulfill in order to use mtDNA analysis in clinics are shown. Stringent methodological criteria of a case-controlled experiment in molecular medicine are indicated. In the third part we suggest, what lessons can be learned for the future and propose guidelines for mtDNA analysis in oncology. Finally we conclude that, although several conceptual and methodological difficulties hinder the research on mitochondrial patho-physiology in cancer cells, this area of molecular medicine should be considered of high importance for future clinical practice.

## The role of mitochondria in carcinogenesis

What is the relationship between mitochondrial dysfunction and carcinogenesis? For many decades mitochondria have been presented only as 'powerhouse' organelles. The primary function of mitochondria, which is ATP production through oxidative phosphorylation (OXPHOS), remained mostly in the focus of interest of biochemists. Nowadays we are becoming more aware of the fact, that these dynamic structures play a pivotal role in cell transformation. Indeed, genetic and metabolic alterations in mitochondria have been shown to be the cause or contributing factors of pathogenesis in a broad range of human diseases, including cancer [1-4]. As already recognized many years ago by Otto Warburg, cancer cells generate excessive lactate in aerobic glycolysis and OXPHOS disruption appears to be a general feature of malignant cells [3,5]. Recent evidence indicates the importance of hypoxia and the progressive elevation in mitochondrial ROS production which over time leads to stabilization of cells via increased HIF-2alpha expression, enabling cells to survive with sustained levels of elevated ROS [6,7]. Recent evidence also indicates that the resulting mutated cancer-causing proteomic feedback amplifiesy cell transformation process by directly affecting mitochondrial function in combinatorial ways and promoting a vicious spiral of malignant cell transformation [8]. Evidence exists that onco-proteins and tumor suppressor proteins physically localize in? to the mitochondria in cancer cells where they directly regulate malignant mitochondrial programs, including apoptosis [7,9]. At the same time the presence of mtDNA mutations in cancer cells have been claimed in a deluge of reports. MtDNA mutations were found in solid tumors, as well as lymphomas and leukaemias [9-13]. Several groups have found associations between somatic mtDNA mutations and cancer development, progression or metastasis [1-3,12,14]. In addition, recent studies have correlated inherited polymorphisms of the mitochondrial genome with the risk for cancer development, including prostate, and oral and colorectal cancer [15,16]. Altogether mitochondrial research offer great promise for the future and seem to offer prominent cancer markers.

We understand that gradually it will be more commonly accepted that mitochondrial medicine is providing better insight into who should receive cancer therapy and what therapy should be administered, ultimately avoiding suboptimal treatment [17-19].

At the same time although many papers have been published in the field of 'mitochondrial oncology', clinicians and patients still cannot fully benefit from the development of molecular mitochondrial research [20-22]. In this article we propose that there are three common problems in the mitochondrial oncology research and that these need to be addressed in any future research. The majority of published work does not offer above all 1) statistically significant data obtained from experiments with clear **methodology**; 2) **cause-effect **explanations (as correlation does not imply causation); 3) **patho-physiological **insight (i.e. what are the functional consequences of specific mtDNA mutations for the cell), and 3) **mechanisms **based explanations. All those deficiencies result in publication of large number of papers which can't be used for cancer diagnostics or prognosis. Above all molecular mechanisms behind mitochondrial carcinogenesis are still uncovered [5,7,22].

## Multiple errors in mtDNA research

Last five years have brought into light that significant number of medical mtDNA studies are based on obviously flawed sequencing results and report phantom mutation [22,23]. A critical revision of the findings reported in previous studies indicated a lack of proper methodological standards that led to an over-interpretation of the role of the mtDNA in cancer development and progression. It was shown that more than half of published mtDNA sequencing studies contain obvious errors, no matter in which journal the investigation is published [22-28]. Oncogenetic contexts of mtDNA mutation cancer reports resulted in conclusion of false association between seemingly causal variants and tumor instability [22,29]. It was the worldwide mtDNA phylogeny analysis that revealed that contamination and sample mix-up episodes were erroneously interpreted as mtDNA instabilities in multiple types of tumors and/or reported as germline mutations, while those were actually mtDNA polymorphisms [22,25]. It is therefore obligatory to remember that tumor sample is apparently distinguished from the corresponding normal tissue sample by **somatic mutations**, while mtDNA variation between different cases, between patients populations or patient sequence and rCRS must be defined as inherited polymorphism [30,31].

In the light of reports from Bandelt et al. one need to remember that although a mitochondrial paradigm of metabolic and degenerative diseases, aging, and cancer was proposed [5], the study of somatic DNA instabilities still constitutes a debatable topic. Different causes can lead to DNA alteration patterns reported between different cells or tissues from the same individual. Patterns of instabilities can arise from technical errors at any stage of the analysis, including DNA extraction, amplification, and sequencing, mutation screening and documentation handling. In particular inadvertent DNA contamination and sample mixing yield mosaic variation that was erroneously interpreted as mutations [23,27,32,33]. The authors claim that the sequencing efforts in the field of cancer were challenged by technical problems, caused also by DNA sequencing biochemical problems, incomplete sequencing, and misdocumentation. Moreover insufficient reference to previously published reports resulted in interpretive problems [34]. Moreober novelty of a given mtDNA variant was most often equated with nonregistration in MITOMAP database - as in the case of multiple mutations of MT-ND3 gene and m.15287T > C [20], which is obvious overestimation since MITOMAP is simply incomplete database in comparison to PubMed. As proven by Bandelt and Salas phylogenetic analysis of whole mitochondrial genomes mutations provides method for elimination of artificial mutations in studies and helps to verify the accuracy of mtDNA analysis [22,23,33].

Finally one need to remember that wrong conclusions concerning the pathogenic status of specific mtDNA mutations may also be influenced by errors in logistics of laboratory work, data handling, and accidental amplification of nuclear mitochondrial pseudogenes (NUMTs) and analysis of their sequences since NUMTs are a potential source of contamination during mitochondrial DNA PCR amplification [35]. Mitochondrial genome disease-associated biomarkers - polymorphisms or mutations - must be rigorously authenticated to preclude any affiliation with paralogous nuclear pseudogenes and requires careful primer design and testing. Direct pseudogene contribution in the analysis is not always obvious and can confound suggested mtDNA biomarkers. Potential markers must be thoroughly investigated to exclude false mtDNA mutations in the interpretation sequencing data. BLAST searches of nuclear pseudogenes eliminate the possibility of integration of these nuclear sequences in mtDNA analysis, since many primers may amplify homologous NUMTs embedded anywhere in the nuclear genome. If the use of mitochondrial DNA analysis, and in particular, somatic mitochondrial genome mutations is of important utility and medical merit, data requires critical follow-up from a pseudogene perspective. For example, mitochondrial PCR protocols may be simultaneously run on nucleic acid recovered from *ρ*^0 ^cells to identify and exclude co-amplification of NUMTs [36]

**Figure 1:**
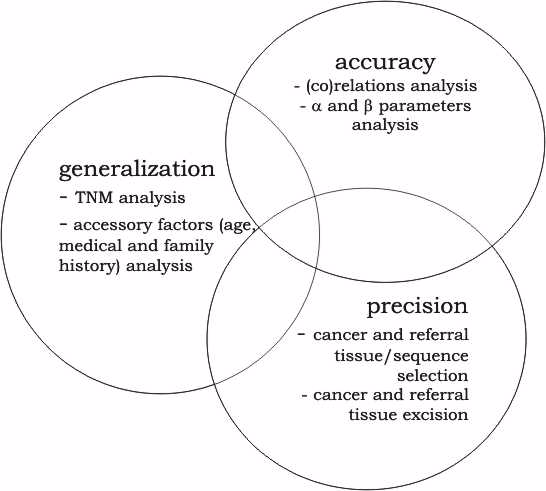
**The three - horn dilemma in molecular mitochondrial marker oncology research**. The figure represents the interdependence of factors that should be considered in mitochondrial oncology experiments design.

With respect to this problem, we underline the fact that while the pattern of mtDNA mutations in cancer tissues may provide markers of potential clinical validity (see Figure 1), numerous published experiments do not fulfill methodological criteria of a case-control molecular-medical experiment and are often more case reports and case studies [22]. At this time many of mtDNA mutation - cancer correlation reports need to be verified with larger sample sizes, as shown by J.A. Califano group [37]. Under these circumstances it is still unclear, how many of the mtDNA mutations reported in cancer cells have been adequately determined [22].

## The challenge of mitochondrial research

To answer the question of mitochondrial role in cancer development, one needs to take into consideration that mutations in mtDNA may result in structural alterations of mitochondrial proteins and disrupted OXPHOS, which in turn would shift the metabolism towards anaerobic respiration - a common feature of cancer cells [38-40]. At the same time, the combination of different defects of the respiratory chain might have substantial physiological effects on the level of production of the carcinogenic reactive oxygen species (ROS) and reactive nitrogen species (RNS) [2,41-43]. Thereupon the disturbance of mitochondrial respiration enhances cell oxidative stress and possibly propels carcinogenic vicious circle [10,14,44]. First of all, molecular research should confirm the biological and physiological significance of mutations, as in the case of reports by Ishikawa et al. [2] and Arnold et al. [45], who have clearly shown the mtDNA sequence influence cell proliferation and cancer metastasis [42,45]. Biochemical approach should be followed by subsequent critical clinical analyses of patient data [46], as in the reports from Zhou et al. [47] and Petros et al. [1].

At the moment is more and more clear that not all mitochondrial mutations reported are actual **causative factors **in cancer development and/or progression, but may also arise as a consequence of cell transformation [48,49]. This paradox leads to a basic chicken-and-egg problem: what is the first cause in cancer development and what is a mere consequence of cell transformation? It seems even more complicated, as often in the case of polymorphisms and mutations the literature contains multiple conflicting reports, regarding which polymorphic base is actually associated with cancer, as it is the case of A10398G [12,50]. This inconsistency makes verification of the mtDNA mutation-cancer correlation more and even more complicated [12,51,52].

To answer the question of mtDNA mutations and polymorphisms involvement in tumor development, detailed biochemical and biophysical analyses of mutation consequences are needed [4,38]. In particular, given the critical role of mitochondria in apoptosis, it is possible that mutations in mtDNA in cancer cells could significantly affect the cellular apoptotic response to anticancer agents and promote multi-drug resistance [14,53-55]. It was proven that frame-shift muatation of NADH dehydrogenase (respiratory complex I) through ROS mediated phosphatidylinositol 3-kinase-Akt/protein kinase C/histone deacetylase pathway may inhibit apoptosis [43,54], including resistance to apoptosis induced by staurosporine, 5-fluorouracil, and cisplatin in vitro [56]. On the second hand recent findings regarding the role of some mitochondria-localized proteins (including p53, AAA+ proteases, or mitochondrial heat shock proteins) in the cell cycle and apoptosis further support the mitochondria - mediated apoptosis resistance hypothesis [9,57-59]. In this case a global analysis of the localization and level of mitochondrial transcripts and proteins might help to determine specific mito-markers [5,8,60]. As mentioned before, empirically observed co-variation is a necessary but not sufficient condition for establishing causal links between mtDNA changes and tumorigenesis. Thus, **multidisciplinary effort **is needed to confirm the significance of mitochondrial failure in cancer evolution [18]. It is because mitochondrial failure has been reported at all levels of their structure and function, including abnormal ultrastructure and deregulation of metabolism [1,9], but signaling pathways leading from mtDNA mutation via mitochondrial transcriptome, proteome to metabolome and to nuclear response and cancer development have not yet been defined [61-63]. It has only been shown that mtDNA mutations observed in tumors result in abnormal expression of mtDNA genes. Now there are still missing links between respiratory deficiency, structural impairment of mtDNA-encoded proteins and specific mtDNA mutations in specific types of tumors. Currently there is very little integration between the research in cell physiology (mitochondrial failure) and in body physiology (tumor development) [11,60]. A holistic understanding of mitochondrial function with a special emphasis on genomic, proteomic, and functional information in mitochondria requires collaborative efforts from multiple disciplines. In particular, efforts coupling computational biology with genomic and proteomic sciences are essential to advance the field. We also need to build new models and experimental tools for assessing mitochondrial biology and to interface with animal models of disease in a reasonably high throughput manner [64]. Holistic models are essential to advance understanding of mitochondrial function in the genomic and proteomic perspective. We also see a need to bridge molecular biology with clinical sciences by developing *in vivo *measurements of mitochondrial function. While animal models and *in silico *models remain indispensable, quantitative markers cannot be created without quantitative measurements of mitochondrial function in humans [65,66]. This requires novel experimental tools and collaboration between basic scientists, clinicians and engineers. Finally, the data obtained in computational, molecular, biochemical and epidemiologic approaches should form the basis for constructing a model of mitochondrial function that will provide insight into all levels of organization and assist in developing mitochondrial medicine. Therefore we believe that greater emphasis should be put on functional research, since the identification of mtDNA mutations in oncology is likely to have a significant impact on clinical and prognostic procedure [3,5,21].

## mtDNA research guidelines

In the light of presented re-analysis of previous experiments a detailed design of new trials is necessary [22]. Nevertheless we believe that global analysis of mtDNA mutation pattern in cancer cells should result in the proposal of 'mito-markers' specific for particular sub-types of cancer [3,11,21]. MtDNA mutation patterns could provide prognostic and/or predictive information about tumors, including the qualification of residual risk of distant recurrence in patients with negative lymph nodes. By virtue of the mtDNA clonal nature and high copy number, the detection of mtDNA mutations may provide a powerful molecular tool for tumor detection with advantages over nuclear genome-based methods, as body fluids or non-invasive tissue access are available for mitochondrial DNA recovery [67]. For that reasons, once a specific, clear and testable hypothesis is stated, every particular correlation study or experiment should recruit additional control groups consisting of either healthy volunteers and/or a second cohort of patients. Moreover, known classical diagnostic and prognostic markers must be considered along with new molecular markers to elucidate independent prognostic factors. Furthermore, sufficient numbers of participants must be enrolled to perform formal power calculations. Only when these additional conditions are fulfilled, the molecular hypothesis and biochemical result may be transferred to clinical settings and translated into patient benefits [9,21,68-70].

**Figure 2:**
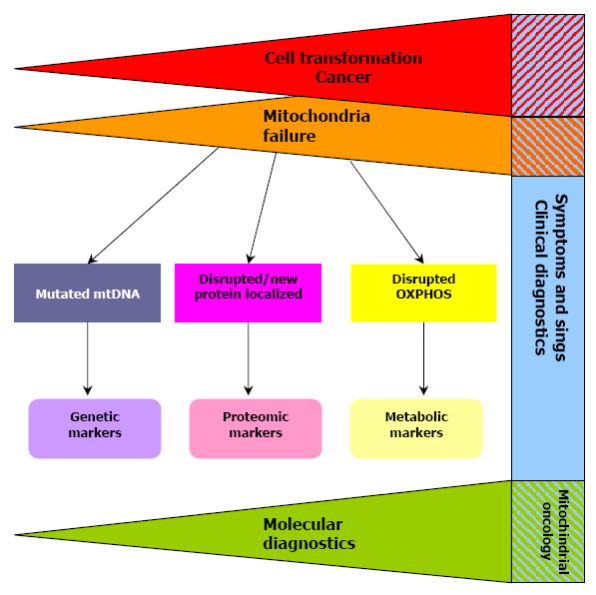
**Possible usage of mitochondrial bio-markers in cancer screening, diagnostics and prognosis**. The co-dependence of mitochondrial failure (orange) and cancer development (red); mitochondrial disruption is accompanied by cell transformation and cancer development (red/orange-blue dashed area). At each stage of tumor growth, mitochondrial markers (purple, pink, yellow) may be used, and provide new tools of clinical utility, hence uniting molecular biology (green) and clinical diagnostics (blue) in 'mitochondrial oncology' (blue-green dashed area).

At the same time if cell biology and molecular phenotype of a particular mutation are known [71,72], strictly controlled experiments on normal and cancer tissue from the same patient, as well as analyses of different cancer tissues acquired from patients with cancer at stage may help to verify the role of this mutation in cancer biology. The significance of mutations and polymorphism in cancer development should also be addressed in controlled populations composed of patients with other TNM and cancer indispensable to confirm medical importance of basic science phenomena (see Figure 2). It is only recently when first prospective cohort study on mtDNA copy number and cancer risk was published [73]. Therefore we would like to stress that only precisely design studies may workout clinically relevant data and resolve the causality dilemma of mtDNA mutations and cell transformation.

Finally, we need to emphasize the role of multidisciplinary approach and close collaboration between basic and clinical scientists in designing and conducting studies aimed at developing and evaluating novel cancer diagnostic methods or therapies [5,9,37]. Only crosstalk of many specialists may help to ensure that fundamental research feeds into clinical practice in ways that benefit patients. Lastly, we would also like to suggest that in order to be able to develop new clinical strategies, it is essential to establish new interdisciplinary training programs in cancer biology. Among other areas, the curriculum of such a program should provide strong background in molecular genetics, experimental methodology as well as human physiology and pathology. Trainees (pre-doctoral and postdoctoral students) should be exposed to a broad range of cancer-related research encompassing both basic and clinical aspects of the disease. Such programs are currently being developed, for example in the Roswell Park Cancer Institute, while funding bodies have recognized a need to support joint basic and clinical training programs.

## Conclusions

It is hard to neglect the importance of mitochondria in cancer biology. Polymorphisms (and mutations) of mtDNA, even driven by random processes during malignant transformation, present an excellent possibility for early tumor detection by analyzing the bodily fluids from patients with tumors [74,75]. New molecular mitochondrial markers might be of pivotal importance as histopathologic subtypes of cancer are associated with distinct clinical manifestations, but their diagnosis is often difficult because some tumor subtypes have overlapping microscopic characteristics. Therefore, ancillary methods are needed to optimize classification and we believe that distinct mitochondrial gene mutation-polymorphism profiles might serve as adequate pathologic markers [76]. In selected cancers the development of mito-marker(s) could provide additional or alternative diagnostic and prognostic tools for oncologists and pathologists. The analysis based on mito-markers could significantly enhance the specificity of cancer detection and prediction of tumor behavior, as well as of patient outcome (see Figure 2) [77]. When established, mitochondrial-markers could help in selecting genetically predisposed subjects, setting early diagnosis [5,11], predicting prognosis, managing follow-up [67] and choosing the best therapeutic approaches [5,53]. Mitochondrial polymorphisms may be candidates for cancer biomarkers, and deserve further investigation, perhaps through the use of experimental models including cybrids and analysis of large cohorts of patients. However, when conducting research it is necessary to develop standard experimental and data analysis procedures in order to validate those biomarkers so that they can be reliably used in several laboratories for cancer prognosis in asymptomatic patients, facilitating diagnosis once symptoms appear, or monitoring individuals known to be at high risk [3,5,14,21]. Quantitative mitochondrial biomarkers may aid histopathological analysis of tumor biopsies for the diagnosis, classification and characterization of the differentiation state of a tumor. There is now a widespread consensus that other prognostic factors, different to those included in the TNM-stages system, are required to improve the accuracy in the management of cancer patients, therefore the development of mitochondrial oncology is to benefit clinical practice. Also carriers of some germ-line mtDNA polymorphism could be more susceptible to cancer development and therefore selected as candidate population for intensive prevention and early detection programs [2,4,12,46]. Lastly mitochondria are also emerging as targets for anti-cancer drugs. Many mitocans selectively interfere with the bioenergetic functions of cancer cell mitochondria, causing major disruptions often associated with ensuing overloads in ROS production leading to the induction of the intrinsic apoptotic pathway [78].

## Competing interests

The authors declare that they have no competing interests.

## Authors' contributions

AMC, JSC, FC, WK - have made substantial contributions to the conception and design of the paper, AMC, AK, WK - have been involved in drafting the manuscript, JSC, AMC - have designed and drawn figures, AS, AK- revised and corrected the manuscript.
